# Correction: Improving on estimates of the potential relative harm to health from using modern ENDS (vaping) compared to tobacco smoking

**DOI:** 10.1186/s12889-022-13983-3

**Published:** 2022-09-21

**Authors:** Nick Wilson, Jennifer A. Summers, Driss Ait Ouakrim, Janet Hoek, Richard Edwards, Tony Blakely

**Affiliations:** 1 University of Otago, Wellington, New Zealand; 2 Population Interventions, Centre for Epidemiology and Biostatistics, Melbourne School of Population and Global Health, University of Melbourne, Melbourne, Australia

**Correction: BMC Public Health 21, 2038 (2021).**

**https://doi.org/10.1186/s12889-021-12103-x**

The corrected Abstract of this article [1] should read as follows:

This corrected Abstract replaces the previous one. The original work attempted to quantify the relative harm to health from electronic nicotine delivery systems (ENDS) compared to smoked tobacco. As such it used various biomarker studies and previously modelled estimates of smoking-related health loss. However, we now consider that the data limitations with the selected studies and the assumptions involved in our method, are too problematic to allow for a valid quantitative assessment. The original manuscript text remains below to show the approach taken, but interested readers are directed to the correction file that explains the specific limitations with the studies and limitations with the analysis. This correction file also details some potential ways in which future studies can help advance knowledge of this topic. Our revised conclusion is therefore that despite our previous analysis, given the data and methods limitations in the biomarker studies we identified, it seems premature to develop quantitative estimates of the relative harm to health from using modern ENDS (vaping) compared to tobacco smoking.

At the request of the Editor, we now outline our reasoning for this more cautious conclusion below.

**1) The identified studies did not address potentially major toxicants involved in vaping (e.g., fine particulates and formaldehyde) and only included biomarker measurements for a small proportion of the likely total number of toxicants.**

As an example, neither of two key toxicants (i.e., fine particulates and formaldehyde [2]) were considered in the five studies we used in the analysis. Another gap of note (due to the lack of data in the five studies) was the lack of inclusion of biomarkers of exposure to potentially toxic metals/metalloids such as cadmium, lead and arsenic. This is despite a systematic review [3], which reported that: “Most metal/metalloid levels found in biosamples of e-cigarette users were similar or higher than levels found in biosamples of conventional cigarette users, and even higher than those found in biosamples of cigar users.”

The biomarkers we used from the five selected studies only included a total of seven biomarkers (3-HPMA, CEMA, HMPMA, NNAL, PheT and COHb/eCO). Yet, tobacco smoke contains over 7000 chemicals, hundreds of which are toxic and around 70 of which are known to cause cancer [4]. Although much fewer than the number present in tobacco smoke, the best estimate we identified was of “over 80” chemicals in vaping/ENDS aerosol [5]. However, this latter figure does not capture the thousands of different vaping product flavourings, some of which may have unique lung-damaging effects [6].

The limited selection of biomarkers available for our analysis, therefore, warrants more caution than we initially expressed in the estimated potential relative health impact of vaping vs smoking.

**2) Incomplete compliance with being an “exclusive vaper” (i.e., no smoking) creating the risk that estimates of toxicant levels and risks of relative harm were too high for this group.**

This was a likely problem with all of the five studies except the confinement study [7], and even that study provided no details of measures taken to completely rule out access to illicit smoking. After the publication of our analysis, one study team (Hatsukami et al. [8]) kindly provided us with additional data on verified non-smoking status that allowed us to explore the issue further. However, drawing on these data markedly reduced the sample size to only 10 subjects who were biochemically verified as not smoking, thus rendering the sample too small to support robust conclusions.

**3) Selected studies were likely to be confounded by extraneous sources of toxicants (e.g., air pollution, secondhand smoke and dietary sources).**

Only one of the five selected studies was a confinement study [7], that included an abstinence comparison group. Whilst subjects were randomised in this confinement study, data on the balance between subjects in important confounders such as acrolein in the diet were not presented. For the other relevant studies, the background exposures of toxicants (e.g., from secondhand smoke, other air pollution or diet) and whether this varied between comparison groups were not documented, and hence it was not possible to assess the degree to which biomarker levels in the smoker and ENDs groups were likely to be due to extraneous sources. We attempted to adjust for background levels of acrolein; however, the study we used as the basis for this adjustment [9], reported data from a time when background air pollution levels and secondhand smoke exposure levels would have been higher than is currently the case, so may have over-estimated exposure among the study participants that was due to extraneous sources. We could not identify robust background exposure data from air pollution and diet for the other six toxicants considered in the five studies. Similarly, the studies measuring carboxyhaemoglobin (COHb) and our analysis did not adjust for *endogenous* production of COHb in the body [10].

**4) Only two of the five identified studies were randomised controlled trials (i.e., of preferable methodological quality).**

Our study included only two randomised controlled trials (RCTs) [7, 8]; the three other studies were observational and therefore of lower methodological quality. It is feasible, for example, that the ENDS and smoker groups in the observational studies differed systematically in their extraneous exposure to toxicants in diet or air pollution, creating bias in the comparison of biomarker levels between the groups. Post-publication, we explored conducting further analyses using only the two RCTs (along with the additional unpublished data provided to us post-publication by Hatsukami et al). However, this approach resulted in findings based on a single industry-funded study [7] and only *n* = 10 subjects (i.e., for biochemically-verified exclusive vapers) from the non-industry funded trial [8]. Furthermore, authors of one of the trials [7] noted some anomalous results for the biomarker NNN that concerned them. Taking into account the other limitations noted, we concluded that using only these two RCTs would not enable us to adequately estimate relative levels of biomarkers and harm in vapers and smokers.

The three observational studies all have the potential problem of confounding from unmeasured and unknown confounders, and the problems of potential non-exclusive vaping (as detailed above). But below we detail some of the more specific potential problems with these studies:

- The study by Boykan et al. [11] involved a narrow age range of subjects (aged 12 to 21 years old), involved a mix of vaping devices, and was a convenience sample of three outpatient clinics (Stony Brook, New York State, USA). The low cotinine levels in exclusive vapers suggested low intensity of ENDS use that might be atypical relative to typical (i.e., more intensive) ENDS use. Hence the biomarker levels for ENDS users in this study may underestimate the true risk.
- Nga et al. [12] drew on a convenience sample (i.e., of staff, supporting staff or patients visiting the Oral Health Center, International Medical University Kuala Lumpur, Malaysia). Participants selected the mix of products studied (ENDS or a heated tobacco product) and the only biomarker data was for eCO, which was collected only hours after participants used the new product.
- Oliveri et al. [13] used a mix of vaping devices (tank-based and cartridge); the study was industry-funded and seems to have had some problems with non-exclusive vaping e.g., “the observed levels of >5% saturation suggests that a select group of AEVP were not exclusive users and may have been smoking cigarettes.”

**5) Some included studies were very short-term.**

Usage patterns among short-term users of ENDS may have differed from those exhibited by more experienced ENDS users, which may have affected their biomarker measurements. This was potentially relevant to the RCT involving only five days of ENDS use [7], or the observational study involving just hours of use [12]. Another problem with such short-term studies is the inadequate washout period for some biomarkers (e.g., for the biomarker NNAL, the half-life in the human body is 10 to 18 days [14]).

**6) Two of the five studies were industry-funded.**

Two of the five selected studies [7, 13] were industry-funded (by JUUL and Altria). Given the evidence that industry-funded studies are more likely to find industry-favourable results (e.g., for ENDS [15] and for the pharmaceutical industry [16]), these arrangements are potentially problematic. We could have excluded these two studies, but doing so would have left only one RCT and two lower quality observational studies.

**7) Vaping device heterogeneity and modernity – impacting external validity.**

Only two of the included studies exclusively involved the more modern cartridge-based ENDS devices [7, 8]. With the growing international market dominance of these cartridge-based products, the relevance of the other studies is diminished.

**8) Potentially different trajectories between smoking and vaping – impacting external validity (especially relevant given the long time courses of diseases such as cancers).**

The five selected studies all represented points in time in the long-term trajectory of ENDS use by individuals and within populations, and include diverse brands and product types (of both ENDS products and comparative tobacco brands). Trajectories of ENDS use and smoking may diverge further in the future. For example, smoked tobacco products have changed little over many decades and it seems likely that many people who smoke will continue smoking long-term at approximately the same intensity and exhibit similar levels of biomarkers, and experience similar levels of harm. However, this outcome seems less certain for ENDS use. ENDS users/vapers may be more or less likely to continue ENDS use long-term compared to people who smoke. There may also be future changes to ENDS technology and usage patterns that affect exposure levels among ENDS users (e.g., based on changes in relative nicotine levels, or potential delineation of smokefree and vapefree areas, or if public tolerance of ENDS increases relative to smoking, or if the design of ENDS products evolves further). This may mean that the biomarker levels observed in ENDS users in the five studies may not represent longer term biomarker levels with continued ENDS use and hence the approach we used to estimating relative harm may be inaccurate.

**9) Various other study limitations.**

In the *Discussion Section* of the published article, we considered additional limitations. These include the limited number of disease categories selected (only four main groupings), the lack of weighting of the different biomarkers within the linked disease categories (due to lack of adequate data), and the likelihood that toxicant exposure and subsequent risk of cardiovascular disease is non-linear. We considered using data on the latter relationship [17], but given the other limitations with the analysis, this additional analytic refinement did not seem justified at the time.

**Potential next steps for progressing the science on estimating the relative harm of vaping vs smoking.**

High quality long-term cohort studies with health outcome measurements should ultimately provide important information on the relative harm of vaping vs smoking. Such studies may also provide earlier provisional information by using biomarkers and physiological measures.

But perhaps the best short-term way to explore this topic is to conduct additional and larger confinement studies where these involve:

(i) a greater number of biomarkers and physiological measures that cover a wider range of toxicant types (as discussed above);
(ii) three randomised study arms (people who switch from smoking to exclusive vaping, people who stay smoking, and people who switch from smoking to neither vaping or smoking);
(iii) adequate time periods to allow for stability in ENDS use behaviours and for adequate wash-out periods for all the key toxicants so that stable levels are achieved;
(iv) verification methods to ensure no dual use (ENDS and smoking) and assessment of other toxicant exposures (air pollution; diet). Although the latter can be largely addressed with the randomisation process, such data may inform interpretation and generalisability (e.g., if the study was performed in a setting with air pollution and where the population consumed food with relatively high acrolein levels);
(v) funding that is fully independent from industry.

Additional information can also come from theoretical studies (e.g., as per: [2]) or “in silico” modelling studies (e.g., as per one on cannabis vaping: [18]).

All such information is urgently required as policy-makers around the world are having to make decisions on vaping and smoking regulation to get the optimal balance between: (i) facilitating access to vaping to assist smokers to quit or reduce harm from ongoing nicotine dependence; and (ii) minimising youth uptake of both vaping and smoking.
