# Supplementary material for: Improving on estimates of the potential relative harm to health from using modern ENDS (vaping) compared to tobacco smoking
Source: BMC Public Health. 2021 Nov 8;21:2038. doi: 10.1186/s12889-021-12103-x (PMC8577029; doi:10.1186/s12889-021-12103-x)
Supplement: Supplementary file 1 — Additional file 1. Supplementary Information for: “Improving on estimates of the relative harm to health from using modern ENDS (vaping) compared to tobacco smoking” [file 12889_2021_12103_MOESM1_ESM.docx]

**Supplementary Information for:**

**“Improving on estimates of the relative harm to health from using modern ENDS (vaping) compared to tobacco smoking”**

Nick Wilson,^1^ Jennifer A Summers,^1^ Driss Ait Ouakrim,^2^ Janet Hoek,^1^ Richard Edwards,^1^ Tony Blakely^2^

**Literature Search Criteria, Database Search Terms and PRSIMA diagram**

We used all the identified biomarkers for tobacco smoking in a FDA Review document [1] (except for those relating to nicotine and tobacco alkaloids), for inclusion in our literature search. The search criteria and specific terms used are shown in Supplementary Tables 1 to 3 below and Supplementary Figure 1 is of the PRISMA diagram of the process. Two reviewers screened one database each. After initial selection based on titles and abstracts, both reviewers compared selected articles and screened full-text articles against the selection criteria. The third reviewer screened the final selection of articles.

**Supplementary Table 1: Literature search criteria (PICO framework)**

| **Inclusion criteria** | |
| --- | --- |
| Population | Exclusive ENDS users |
| Intervention | Measure of relevant toxicant/biomarker in either urine, blood or exhaled air |
| Comparators | Exclusive tobacco smokers |
| Outcome | **“Carbon monoxide” (“CO”);**  “Carboxyhaemoglobin” (used to measure CO in blood)  **“Tobacco-specific nitrosamines” (“TSNAs”)**  Nicotine-derived nitrosamine ketone (“NNK”)  N-nitrosonornicotine (“NNN”) and its glucuronides (NNN-Gluc)  “NNAL”, the primary metabolite of NNK (the most widely investigated TSNA biomarker)  Nitrosoanabasine (“NAB”)  N'-nitrosoanatabine (“NAT”)  **“Polycyclic aromatic hydrocarbons” (“PAHs”)**  “Pyrene”  “Fluorene”  “Phenanthrene”  “Naphthalene”  **“Volatile organic compounds”** (“VOCs”)  2,5-“dimethylfuran”  “Benzene”  “Toluene”  “Ethylbenzene”  “Xylene”  “Styrene”  “Acrolein”  3-hydroxypropylmercapturic acid (3-“HPMA”) (a mercapturic acid metabolite of acrolein)  “Crotonaldehyde”  “Butadiene”  “Benzene”  “Acrylonitrile”  “Acrylamide”  “Ethylene oxide”  **“Aromatic amines” and “heterocyclic amines”**  3-aminobiphenyl and 4-“aminobiphenyl”  “Dimethylanilines”  **“Metals”**  “Lead” (“Pb”)  “Cadmium” |
| Language restrictions | None |
| Search dates | Restricted to literature published after 1 January 2017, due to development of e-cigarette devices |
| **Exclusion criteria** | |
| Population | Non-Human based studies, dual users of ENDS and tobacco |
| Study design | Case Studies |

**Supplementary** **Table 2: Database Search Terms for PubMed (**Search date 1 September 2020)

| **ID** | **Search** | **Hits** |
| --- | --- | --- |
| #1 | Carbon monoxide OR "CO" OR Carboxyhaemoglobin | 889,062 |
| #2 | "Tobacco-specific nitrosamin*" OR TSNA | 876 |
| #3 | "Nicotine-derived nitrosamine ketone" OR NNK | 1,413 |
| #4 | N-nitrosonornicotine OR NNN OR glucuronid* OR NNN-N-Gluc* | 48,961 |
| #5 | NNAL OR methylnitrosamino | 1,476 |
| #6 | Nitrosoanabasine OR NAB | 5,579 |
| #7 | N'-nitrosoanatabine OR N-nitrosoanatabine OR NAT | 207,991 |
| #8 | "Polycyclic aromatic hydrocarbon*" OR PAH OR Pyrene OR Fluorene OR Phenanthrene OR Naphthalene | 306,959 |
| #9 | "Volatile organic compound*" OR VOC | 18,799 |
| #10 | "2,5-dimethylfuran" OR dimethylfuran OR DMF | 17,546 |
| #11 | Benzene OR Toluene OR Ethylbenzene OR Xylene OR Styrene OR Acrolein | 104,255 |
| #12 | "3-hydroxypropylmercapturic acid" OR 3-HPMA OR HPMA | 1,028 |
| #13 | Crotonaldehyde OR Butadiene OR Benzene OR Acrylonitrile OR Acrylamide OR "Ethylene oxid*" | 82,155 |
| #14 | "Aromatic amine*" OR "heterocyclic amine*" | 5,995 |
| #15 | 3-aminobiphenyl OR 3-Aminobiphenyl OR Aminobiphenyl OR Dimethylaniline* | 2,867 |
| #16 | Metals or “metal and lead” OR "Pb" OR Cadmium OR "Cd" | 1,573,488 |
| #17 | #1 OR #2 OR #3 OR #4 OR #5 OR #6 OR #7 OR #8 OR #9 OR #10 OR #11 OR #12 OR #13 OR #14 OR #15 OR #16 | 3,014,484 |
| #18 | ENDS OR vape* OR vapi* OR vapo* OR e-cig* | 132,178 |
| #19 | tobacco OR nicotine OR smok* | 407,192 |
| #20 | "2017/01/01"[Date - Publication] : "2020/08/25"[Date - Publication] | 4,590,129 |
| #21 | #17 AND #18 AND #19 AND #20 | 475 |

**Supplementary** **Table 3: Google Scholar searches** (search date 1 September 2020. Restricted to articles published between 2017 and 2020 and sorted by relevance. The first ten pages of each search was assessed due to the large number of hits).

| **ID** | **Search** | **Hits** |
| --- | --- | --- |
| #1 | (Carbon OR monoxide OR Carboxyhemoglobin OR Carboxyhaemoglobin) AND ((tobacco OR nicotine OR smoke OR smoking) AND (ENDS OR vape OR vaper OR vaping OR vaporised OR vaporised OR e-cig OR e-cigarette)) | 23,200 |
| #2 | (Tobacco-specific OR nitrosamine OR TSNA) AND ((tobacco OR nicotine OR smoke OR smoking) AND (ENDS OR vape OR vaper OR vaping OR vaporised OR vaporised OR e-cig OR e-cigarette)) | 3,600 |
| #3 | (Nicotine-derived nitrosamine ketone OR NNK) AND ((tobacco OR nicotine OR smoke OR smoking) AND (ENDS OR vape OR vaper OR vaping OR vaporised OR vaporised OR e-cig OR e-cigarette)) | 257 |
| #4 | (N-nitrosonornicotine OR NNN OR glucuronide OR NNN-N-Gluc) AND ((tobacco OR nicotine OR smoke OR smoking) AND (ENDS OR vape OR vaper OR vaping OR vaporised OR vaporised OR e-cig OR e-cigarette)) | 9,830 |
| #5 | (NNAL OR methylnitrosamino) AND ((tobacco OR nicotine OR smoke OR smoking) AND (ENDS OR vape OR vaper OR vaping OR vaporised OR vaporised OR e-cig OR e-cigarette)) | 20,800 |
| #6 | (Nitrosoanabasine OR NAB) AND ((tobacco OR nicotine OR smoke OR smoking) AND (ENDS OR vape OR vaper OR vaping OR vaporised OR vaporised OR e-cig OR e-cigarette)) | 3,370 |
| #7 | (N'-nitrosoanatabine OR N-nitrosoanatabine OR NAT) AND ((tobacco OR nicotine OR smoke OR smoking) AND (ENDS OR vape OR vaper OR vaping OR vaporised OR vaporised OR e-cig OR e-cigarette)) | 21,200 |
| #8 | ("Polycyclic aromatic hydrocarbon" OR PAH OR Pyrene OR Fluorene OR Phenanthrene OR Naphthalene) AND ((tobacco OR nicotine OR smoke OR smoking) AND (ENDS OR vape OR vaper OR vaping OR vaporised OR vaporised OR e-cig OR e-cigarette)) | 15,700 |
| #9 | ("Volatile organic compound*" OR VOC) AND ((tobacco OR nicotine OR smoke OR smoking) AND (ENDS OR vape OR vaper OR vaping OR vaporised OR vaporised OR e-cig OR e-cigarette)) | 9,420 |
| #10 | (2,5-dimethylfuran OR dimethylfuran OR DMF) AND ((tobacco OR nicotine OR smoke OR smoking) AND (ENDS OR vape OR vaper OR vaping OR vaporised OR vaporised OR e-cig OR e-cigarette)) | 4,120 |
| #11 | (Benzene OR Toluene OR Ethylbenzene OR Xylene OR Styrene OR Acrolein) AND ((tobacco OR nicotine OR smoke OR smoking) AND (ENDS OR vape OR vaper OR vaping OR vaporised OR vaporised OR e-cig OR e-cigarette)) | 17,000 |
| #12 | (3-hydroxypropylmercapturic OR 3-HPMA OR HPMA) AND ((tobacco OR nicotine OR smoke OR smoking) AND (ENDS OR vape OR vaper OR vaping OR vaporised OR vaporised OR e-cig OR e-cigarette)) | 314 |

**Supplementary Figure 1: PRISMA diagram [2] for the literature search**

Studies identified through PubMed (n = 475) and Google Scholar first 10 pages search (n = 120)

**Screening**

**Included**

**Eligibility**

**Identification**

Studies screened
PubMed and Google Scholar (n = 584)

Irrelevant studies excluded
(n = 532)

Full-text articles assessed for eligibility (n = 52)

Studies excluded (n = 47)

i) Did not compare exclusive smoking with exclusive ENDS use (n = 8)

ii) Did not involve data collected before 1 January 2017 (n = 20)

iii) Other (n = 19)

Studies meeting inclusion criteria (n = 5)

Duplicate studies excluded
(n = 11)

Additional studies identified through reference list review and published reviews (n = 0)

**Supplementary Table A4: Additional details on the five identified studies used in the analysis**

| **Study** | **Dates of data collection** | **ENDS products used by participants** | **Additional details and comments** |
| --- | --- | --- | --- |
| Boykan et al 2019 [3] | April 2017 to April 2018 | Mainly “pods” (78% of past-week daily users) | This was a cross-sectional study comparing exclusive tobacco users and exclusive e-cigarette users (albeit with no data on previous smoking status in the latter group). It involved those aged 12 to 21 years old and was a convenience sample of 3 outpatient clinics (Stony Brook, New York State, USA). The very much lower urinary cotinine levels in exclusive e-cigarette users vs exclusive smokers suggest low levels of unreported dual use in the e-cigarette users. However, these low cotinine levels also suggest low intensity of ENDS use that might be atypical relative to typical (and more intensive) ENDS use. Hence the biomarker levels for ENDS users in this study may be an underestimate of the true risk. A particular strength of this study was that it collected both self-reported marijuana use (ever, past 30 days, past 7 days) and the associated urinary biomarker data (tetrahydrocannabinolic acid: THCA). As a result, our analyses could use the results from those smokers and ENDS users who were not also marijuana users. |
| Hatsukami 2020 [4] | Study was initiated in 2014 but with the last  follow-up on 2 December, 2018 | “Vuse Solo” | This study was a randomised controlled trial (RCT) in a US adult daily smoker population. Participants were randomized to different products, including one arm to complete substitution of cigarettes with ENDS. “Participants were incentivized for protocol compliance”. The biomarker results were based on the 8 week point. |
| Jay et al 2020 [5] | Between February and July 2018 | The JUUL nicotine-salt pod system (4 flavours: Virginia Tobacco; mint;  mango; and crème) | This was a “randomized, open-label, parallel-cohort, confinement study of healthy adult smokers”. Randomisation was to 4 different flavours of e-cigarettes, to continuation of usual brand, or to cigarette abstinence. The population was based in Lincoln, New England, USA. This study was “sponsored by JUUL Labs, Inc.,” (the manufacturer of the ENDS used in the study). Due to the short study time (5 days) we did not include the results for NNAL (owing to the long half-life of this biomarker – see main text). |
| Nga et al 2020 [6] | Not stated but the year on the grant number and on the ethics application was “2019” | Aspire AVP AIO Kit 700 mAh battery having a 1.2 X coil with e-liquid “Liquideo Evolution” | This was a “quasi experimental study” with convenience sampling (i.e., of staff, supporting staff or patients visiting the Oral Health Center, International Medical University Kuala Lumpur, Malaysia). Participants (all smokers at 10+ cigarettes per day for 5 years) were allowed to select the products being studied (ENDS or a heated tobacco product). Most participants were male (87%) and of Chinese ethnicity (51%). Participants were instructed to remain abstinent from cigarette smoking 12 hours before the experiment (and this was verified with eCO measurement). |
| Oliveri et al 2020 [7] | Between January 2017 and June 2017 | “Own brand of EVP” (e-vapour product). Results indicated 70 used tank-based products and 62 used cartridge-based products | This was a cross-sectional, observational study. Participants were 30–65 years of age. Recruitment focused on the “four regions defined by the US Census Bureau to provide a geographically diverse study population.” The study was conducted by employees of Altria Client Services LLC (Altria is a tobacco company). “Participants were instructed to use their own brand of EVP or conventional cigarettes ad libitum throughout study duration (approximately 30 days from electronic consent and online questionnaire completion to biological specimen collection).” The exclusive ENDS users “identified themselves as former smokers (minimum of 10 cigarettes per day for at least 10 years) and were exclusively using EVPs for at least 6 months and no other tobacco- or nicotine-containing products during that period.” Exclusive adult smokers were “individuals who currently smoked 10 or more conventional cigarettes and have smoked 10 or more cigarettes per day for at least 10 years and did not use other tobacco or nicotine-containing products (including EVPs) in the past 30 days.” |

**Supplementary Table A5: Identified studies of acrolein (3-HPMA) in non-smokers and smokers of relevance to estimating the typical intake derived from non-smoking sources (ordered by decreasing number of study participants as bracketed)**

| **Study** | **Level in non-smokers [A]** | **Level in smokers [B]** | **% of [A] relative to [B]** | **Details** |
| --- | --- | --- | --- | --- |
| Alwis et al 2015 [8] | 219 (n=2296) | 1089 (n=570) | 20.1% | Median levels in μg/g creatinine. From NHANES survey data for the USA. Given the size of this study it was the relative value from this that we used for the calculations in Table 1. |
| Scherer et al 2007 [9] | 337 (n=100) | 1297  (n=194) | 26.0% | Mean levels in μg/24h. Involved separate groups. The study was financially supported by a subsidiary of the German cigarette manufacturers’ association. |
| Frigerio et al 2020 [10] | 160.6 (n=38) | 1301.2 (n=22) | 12.3% | Median levels in μg/g creatinine. Involved separate groups. This was a study of an occupational group in an industry with exposure to volatile chemicals. |
| Carmella et al 2009 [11] | 1500 (n=17) | 10,020 (n=17) | 15.0% | Mean levels in nmol/24h at day 56 after quitting smoking (i.e., the same group of individuals was studied). |
| D’Ruiz et al 2016 [12] | 228.8 (n=13) | 2004.1 (n=13) | 11.4% | Mean levels in μg/24h. Involved the same group – at start of study and after 5 days of abstinence. This study was funded by a fully owned subsidiary of Imperial Brands plc, and the manufacturer of the ENDS used in this study. |
| Lorkiewicz et al 2019 [13] | 294.3 (n=12) | 544.7  (n=8) | 54.0% | Mean levels in (ng/mg creatinine). Involved separate groups. Of note was that this study used first generation “cig-a-likes”. |

**References**

1. Chang CM, Edwards SH, Arab A, Del Valle-Pinero AY, Yang L, Hatsukami DK. Biomarkers of Tobacco Exposure: Summary of an FDA-Sponsored Public Workshop. Cancer Epidemiol Biomarkers Prev*.* 2017;26(3):291-302.

2. Page MJ, McKenzie JE, Bossuyt PM, Boutron I, Hoffmann TC, Mulrow CD, et al. The PRISMA 2020 statement: An updated guideline for reporting systematic reviews. Int J Surg*.* 2021;88:105906.

3. Boykan R, Messina CR, Chateau G, Eliscu A, Tolentino J, Goniewicz ML. Self-Reported Use of Tobacco, E-cigarettes, and Marijuana Versus Urinary Biomarkers. Pediatrics*.* 2019;143(5).

4. Hatsukami DK, Meier E, Lindgren BR, Anderson A, Reisinger SA, Norton KJ, et al. A Randomized Clinical Trial Examining the Effects of Instructions for Electronic Cigarette Use on Smoking-Related Behaviors and Biomarkers of Exposure. Nicotine Tob Res*.* 2020;22(9):1524-1532.

5. Jay J, Pfaunmiller EL, Huang NJ, Cohen G, Graff DW. Five-Day Changes in Biomarkers of Exposure Among Adult Smokers After Completely Switching From Combustible Cigarettes to a Nicotine-Salt Pod System. Nicotine Tob Res*.* 2020;22(8):1285-1293.

6. Nga JDL, Hakim SL, Bilal S. Comparison of End Tidal Carbon Monoxide Levels between Conventional Cigarette, Electronic Cigarette and Heated Tobacco Product among Asiatic Smokers. Subst Use Misuse*.* 2020;55(12):1943-1948.

7. Oliveri D, Liang Q, Sarkar M. Real-World Evidence of Differences in Biomarkers of Exposure to Select Harmful and Potentially Harmful Constituents and Biomarkers of Potential Harm Between Adult E-Vapor Users and Adult Cigarette Smokers. Nicotine Tob Res*.* 2020;22(7):1114-1122.

8. Alwis KU, deCastro BR, Morrow JC, Blount BC. Acrolein Exposure in U.S. Tobacco Smokers and Non-Tobacco Users: NHANES 2005-2006. Environ Health Perspect*.* 2015;123(12):1302-1308.

9. Scherer G, Engl J, Urban M, Gilch G, Janket D, Riedel K. Relationship between machine-derived smoke yields and biomarkers in cigarette smokers in Germany. Regul Toxicol Pharmacol*.* 2007;47(2):171-183.

10. Frigerio G, Mercadante R, Campo L, Polledri E, Boniardi L, Olgiati L, et al. Urinary biomonitoring of subjects with different smoking habits. Part I: Profiling mercapturic acids. Toxicol Lett*.* 2020;327:48-57.

11. Carmella SG, Chen M, Han S, Briggs A, Jensen J, Hatsukami DK, et al. Effects of smoking cessation on eight urinary tobacco carcinogen and toxicant biomarkers. Chem Res Toxicol*.* 2009;22(4):734-741.

12. D'Ruiz CD, Graff DW, Robinson E. Reductions in biomarkers of exposure, impacts on smoking urge and assessment of product use and tolerability in adult smokers following partial or complete substitution of cigarettes with electronic cigarettes. BMC Public Health*.* 2016;16:543.

13. Lorkiewicz P, Riggs DW, Keith RJ, Conklin DJ, Xie Z, Sutaria S, et al. Comparison of Urinary Biomarkers of Exposure in Humans Using Electronic Cigarettes, Combustible Cigarettes, and Smokeless Tobacco. Nicotine Tob Res*.* 2019;21(9):1228-1238.
